# A Polysaccharide Purified From *Ganoderma lucidum* Acts as a Potent Mucosal Adjuvant That Promotes Protective Immunity Against the Lethal Challenge With Enterovirus A71

**DOI:** 10.3389/fimmu.2020.561758

**Published:** 2020-09-29

**Authors:** Yu-Li Lin, Chiaho Shih, Pei-Yun Cheng, Chiao-Li Chin, An-Ting Liou, Po-Yi Lee, Bor-Luen Chiang

**Affiliations:** ^1^Department of Medical Research, National Taiwan University Hospital, Taipei, Taiwan; ^2^Institute of Biomedical Sciences, Academia Sinica, Taipei, Taiwan; ^3^Graduate Institute of Medicine, Kaohsiung Medical University, Kaohsiung, Taiwan; ^4^Graduate Institute of Immunology, College of Medicine, National Taiwan University, Taipei, Taiwan; ^5^Drug Development and Value Creation Research Center, Kaohsiung Medical University, Kaohsiung, Taiwan; ^6^Good Health Food Co., Ltd., Taipei, Taiwan; ^7^Department of Pediatrics, National Taiwan University Hospital, Taipei, Taiwan

**Keywords:** adjuvant, Enterovirus A71, IFN-γ, IL-17, intranasal, mucosal vaccine, polysaccharide from *Ganoderma lucidum*

## Abstract

Enterovirus A71 (EV-A71), the pathogen responsible for the seasonal hand-foot-and-mouth epidemics, can cause significant mortality in infants and young children. The vaccine against EV-A71 could potentially prevent virus-induced neurological complications and mortalities occurring due to the high risk of poliomyelitis-like paralysis and fatal encephalitis. It is known that polysaccharide purified from *Ganoderma lucidum* (PS-G) can effectively modulate immune function. Here, we used PS-G as an adjuvant with the EV-A71 mucosal vaccine and studied its effects. Our data showed that PS-G-adjuvanted EV-A71 generated significantly better IgA and IgG in the serum, saliva, nasal wash, bronchoalveolar lavage fluid (BALF), and feces. More importantly, these antibodies could neutralize the infectivity of EV-A71 (C2 genotype) and cross-neutralize the B4, B5, and C4 genotypes of EV-A71. In addition, more EV-A71-specific IgA- and IgG- secreting cells were observed with the used of a combination of EV-A71 and PS-G. Furthermore, T-cell proliferative responses and IFN-γ and IL-17 secretions levels were notably increased in splenocytes when the EV-A71 vaccine contained PS-G. We also found that levels of IFN-γ and IL-17 released in Peyer’s patch cells were significantly increased in EV-A71, after it was combined with PS-G. We further demonstrated that both CD4^+^ and CD8^+^ T cells were able to generate IFN-γ and IL-17 in the spleen. An easy-accessed model of hybrid hSCARB2^+^/^+^/stat-1^–^/^–^ mice was used for EV-A71 infection and pathogenesis. We infected the mouse model with EV−A71, which was premixed with mouse sera immunized with the EV-A71 vaccine with or without PS-G. Indeed, in the EV-A71 + PS-G group, the levels of VP1-specific RNA sequences in the brain, spinal cord, and muscle decreased significantly. Finally, hSCARB2-Tg mice immunized via the intranasal route with the PS-G-adjuvanted EV-A71 vaccine resisted a subsequent lethal oral EV-A71 challenge. Taken together, these results demonstrated that PS-G could potentially be used as an adjuvant for the EV-A71 mucosal vaccine.

## Introduction

Enterovirus type A71 (EV-A71) has become the main cause of epidemics such as hand-foot-and-mouth disease (HFMD), acute flaccid paralysis, brainstem encephalitis, and aseptic meningitis, and is associated with severe fatal neurological disorders in infants and children (under 5 years of age) (1–4). EV-A71 is a member of the *Picornaviridae* family, of the human enterovirus A species. EV-A71 is a non-enveloped virus with a single-stranded RNA genome (4–6). EV-A71-associated HFMD outbreaks have been reported worldwide, and hundreds of children have died from serious encephalomyelitis-related complications (2, 4, 7, 8). It was the first large-scale HFMD outbreak in the Asia-Pacific region that caused 1.5 million infections; in 1998, a total of 405 severe cases (including 78 deaths) were reported during the epidemic of HFMD in Taiwan (9). In China, there were as many as 2 million cases of HFMD infections, and 129 deaths were reported in 2005. Presently, there are no effective antiviral drugs for treating EV-A71 infections. The precaution measures for the EV-A71 epidemics can only depend on public surveillance.

Human scavenger receptor class B, member 2 (hSCARB2) has been identified as a cellular receptor for EV-A71 (10, 11). SCARB2 is widely expressed in several human tissues and cells, including neurons in the central nervous system (CNS) (12). Mouse cells transformed with hSCARB2 are sensitive to all EV-A71 strains (13, 14), and exhibit greater virus binding, internalization, and uncoating (15); however, mouse SCARB2 does not act as a receptor for EV-A71. hSCARB2-Tg mice are susceptible to EV-A71 infection (16). hSCARB2-Tg mice infected with EV-A71 exhibit symptoms of neurological disease considerably similar to those of EV-A71 encephalomyelitis in humans. Therefore, it is a useful model for the study of EV-A71 pathogenesis and for the development of vaccines and antiviral drugs.

Due to the hidden risk of a CNS infection, an effective vaccine against EV-A71 could prevent the morbidity and mortality caused by the virus. In recent years, alum-adjuvanted and inactivated EV-A71 vaccines have been approved in China (17). However, immunization with EV-A71 vaccines was performed via the intramuscular route, which is not an ideal administration route for children. Here, we aimed to develop a safe, needle-free vaccine for mucosal delivery in young children. Mucosal vaccines could efficiently induce secretory IgAs on mucosal surfaces, thereby preventing and limiting infections in the virus entry site (18). Only a few mucosal vaccines are commercially available at present. While developing vaccines, adjuvants are often used to stimulate mucosal vaccines, to enhance the immunity and the effects of mucosal vaccines.

The use of appropriate adjuvants has generated significant interest in intranasal vaccination, because of their ability to establish a strong mucosal immune response, and cause little pain and dread, specifically in newborns and children (19). An perfect mucosal adjuvant can induce mucosal antibody secretion and antigen residence time, and decrease the required antigen dose. Due to their high toxicity and clinical side effects, the use of previously developed mucosal adjuvants, such as cholera toxin and heat labile toxins of Escherichia *coli*, has been hindered (20). Thus, we aim to design and produce a new generation of adjuvants. While designing new adjuvants, their effects on dendritic cells (DCs) need to be considered, because DCs play an important role in both the innate and adaptive immune systems. Adjuvants could increase the expression of presenting and costimulatory molecules on DCs, such as CD80, CD86, and MHC II, and increase the secretion of cytokines (such as IFN-γ and IL-2) (21, 22). Additionally, Toll-like receptors (TLRs) are located extracellularly on the cell membrane or intracellularly in the endosome or cytosol (23). Participation usually causes the production of cytokines and chemokines, thereby promoting the recruitment and activation of APCs, and subsequently triggering adaptive B cell and T cell responses. TLR ligands can be used as both systematic and mucosal adjuvants.

*Ganoderma lucidum* is a medicinal mushroom from China. It has been widely used for promoting health in Asian. *G. lucidum* was reported effective in regulating immune functions and inhibiting tumor growth (24, 25). The structure of polysaccharide purified from *G. lucidum* is a branched (1→6)-β-D-glucan, which contains a backbone chain of (1→3)-linked D-glucose residues (24). Some reports have shown that *G. lucidum* has an anti-tumor effects, which were attributed to the activation of the host immune response (26). Recently, Su et al. found that extract from the spores of *G. lucidum* would serve as a antitumor adjuvant via a restoration on the exhausted (27). In 2005 and 2006, we demonstrated that PS-G could effectively accelerate the activation and maturation of immature DCs favoring a T helper 1 immune response (28, 29). We found that PS-G could induce innate immune responses via TLR4 activation. PS-G has also been shown to induce Th1 immune responses, characterized by IFN-γ secretion and IgG2a generation in the mouse (29). Here, we administered the EV-A71 mucosal vaccine with PS-G as an adjuvant, via the nasal route, and studied its effects. We demonstrated that PS-G adjuvanted EV-A71 induced a potent immune response to EV-A71 virions and against EV-A71 infections.

## Materials and Methods

### Viruses and Vaccines

The EV-A71 strain TW/2272/98, which was used to obtain the purified, inactivated EV-A71 vaccine, was generously supplied from Prof. Shin-Ru Shih (at the Research Center for Emerging Viral Infections, College of Medicine, Chang Gung University, Taiwan). EV-A71 strains 200307025 (B4 genogroup, isolated in 2003), 20080738 (B5 genogroup, isolated in 2008), and 200802571 (C4 genogroup, isolated in 2008) were obtained from the Centers for Disease Control, Taiwan. EV-A71 TW/4643/MP4 (MP4, genotype C2; the fourth generation of mice-adapted EV-A71 from EV-A71 TW/4643/98), which caused increased virulence in mice, was generously supplied from Prof. Jen-Ren Wang (at the National Cheng Kung University, Taiwan) (30). EV-A71 viruses were propagated in rhabdomyosarcoma (RD) cells (ATCC CCL-136) cultured in Minimum Essential Medium Alpha (α-MEM, HyClone^®^) at 37°C. The virus was inoculated into RD cells and allowed to grow for 3 days, the supernatant was harvested and centrifugated at 3200 *g* for 15 min. Subsequently, the collected viral supernatant was further treated with benzonase at 37°C for 1 h. Then, the viral supernatant was concentrated 20–25-fold by a tangential flow filtration system, using a hollow fiber with a molecular weight cut off (MWCO) of 300 kDa, under a transmembrane pressure maintained at ∼10–15 psi. The concentrate was diafiltrated with the exchange buffer, consisting of 50 mM sodium citrate (pH 6.4) and 0.15 M NaCl before being subjected to fractionation on a Sephacryl s-400 (26/60) column in an AKTA FPLC (fast protein liquid chromatography) system with phosphate buffer saline (PBS) being used as the elution buffer in a flow rate of 1.3 ml/min. Virus-containing fractions were collected, filtered, and concentrated via a centricon filter (MWCO 100 kDa). Purified EV-A71 viruses were inactivated with 1/4000 formalin at 37°C for 24 h. To confirm the complete loss of infectivity of inactivated EV-A71 viruses used for vaccinating mice, we performed the 50% tissue-culture infectious dose (TCID_50_) assay and ensured that they caused no cytopathic effects in RD cells for at least 7 days.

### Preparation and Analysis of PS-G

Based on the protocol followed in our previous study (26), the fruiting bodies of *G. lucidum* were washed, decomposed, and extracted using boiling double-distilled water (95–100°C) for 12 h. The hot water extract of *G. lucidum* was concentrated using a vacuum rotary evaporator, precipitated with ethanol, and separated into polysaccharide (insoluble in alcohol) and non-polysaccharide fractions (soluble in alcohol). Next, the obtained crude polysaccharide was subjected to gel filtration in a Sephadex G 50 column (Pharmacia, Uppsala, Sweden), and purified via anion exchange chromatography, using a diethylaminoethyl cellulose column (24). The purified PS-G was lyophilized and stored at 4°C. As for the concentration of PS-G, the assay is based on the dry weight of the PS-G powder. The polysaccharide concentration in PS-G was determined using the phenol-sulfuric acid method (31), and protein detection was performed using the BCA protein kit (Thermo Scientific Pierce, United States). The results showed that PS-G is a protein-binding polysaccharide composed of 95% polysaccharides and 5% proteins. The monosaccharide composition analysis was performed using the polysaccharide component assay kit (32) from SugarLighter (Taiwan) by NMR (33), which revealed that PS-G was primarily composed of glucose (79%) and mannose (21%; Supplementary Figure 1 in Supplementary Material). The molecular weight (MW) of PS-G determined by diffusion-ordered NMR spectroscopy (DOSY NMR) (34) by SugarLighter (Taiwan) was approximately 15 kDa (Supplementary Figure 2 in Supplementary Material). To avoid the contamination of PS-G by LPS, we measured the LPS content using the chromogenic Limulus amebocyte lysate assay. The results showed that there was no detectable endotoxin in the PS-G samples (<0.10 endotoxin units/ml).

### Mouse Immunization

Specific-pathogen-free female C57BL/6 mice (6-week-old) were used to study the effect of PS-G as an adjuvant on the immune response to EV-A71. Six mice in each group were intranasally immunized with 12 μl (6 μl per nostril) of the vaccine, including RD lysate, 2.5 μg of formalin-inactivated EV-A71, and 2.5 μg of formalin-inactivated EV-A71 plus 20 μg of PS-G as an adjuvant; the mixture was slowly dropped into each nostril to prevent suffocation. The uninfected RD lysate was subjected to the same purification processes as formalin-inactivated EV-A71 (propagated in RD cells), and used as a negative control. Mice were inoculated three times, on day 0, 21, and 42. Blood samples were collected at 2 weeks after the third immunization process and stored at −80°C until further use. Animal experiments were approved by the Institutional Animal Care and Use Committee (IACUC) at the College of Medicine, National Taiwan University (Taipei, Taiwan), and performed in accordance with approved guidelines.

### Sample Collection

Saliva samples were collected from mice via the intraperitoneal administration of 100 μg pilocarpine hydrochloride (Sigma-Aldrich, St. Louis, MO, United States). Saliva samples were then centrifuged for 10 min at 12000 rpm, and supernatants were collected. For nasal wash collection, the supramaxilla and submaxilla of mice were surgically separated, and the nasal cavity was washed with PBS (0.5 ml). To collect bronchoalveolar lavage fluid (BALF), the trachea of mice was surgically exposed and intubated, and the lung was lavaged three times with PBS (0.5 ml). Subsequently, nasal wash and BALF samples were centrifuged for 5 min at 360 *g*, and supernatants were collected for antibody detection. To obtain feces extracts, fresh excrements of mice were collected and their concentration was adjusted with PBS to 100 mg/ml; samples were agitated for 15 min. Then, the scattered feces were centrifuged for 10 min at 27,000 *g*. Supernatants were collected and centrifuged again. After centrifugation, supernatants were collected as feces extracts for antibody detection via enzyme-linked immunosorbent assay (ELISA).

### Detection of EV-A71-Specific Antibodies

As shown in our previous study (35), microplates (Nunc) were coated overnight with inactivated EV-A71 and blocked with TBST-1% BSA. Then, serum, saliva, nasal wash, BALF, or feces extract were added and incubated for 2 h. Subsequently, plates were washed and the HRP-conjugated goat anti-mouse IgG (1:10000, Bethyl Laboratories, Inc.) or anti-mouse IgA (1:5000, Bethyl Laboratories, Inc.) was added. Then, 100 μl of 3, 3’, 5, 5’-tetramethylbenzidine was added, and color was developed in the dark for 20 min. We added 1 M H_2_SO_4_ to stop the reaction. The plate was detected using the SpectraMax M5 Multi-Mode Microplate Reader (Molecular Devices) at 450 nm and 550 nm. Results were expressed in ELISA units (EU): EU = (A_sample_ – A_blank_)/(A_positive_ – A_blank_).

### Detection of Neutralization Titers

The serum was heat-inactivated for 30 min at 56°C, after which 50 μl of 2-fold diluted serum was mixed with 50 μl of 100-fold TCID_50_ EV-A71 in a 96-well plate, and incubated for 1 h at 37°C (35). Then, a mixture containing 2 × 10^4^ RD cells in 100 μl of α-MEM-2% FBS was added to the mixture, and its cytopathic effects was determined after 4 days. The maximum dilution that resulted in no cytopathic effects was identified as its neutralization titer.

### Antibody-Secreting Cell Analysis

According to our previous study (35), Enzyme-linked immunospot (ELISPOT) was used to detect EV-A71-specific IgG- and IgA-secreting cells. We coated 96-well microplates (Millipore, United States) with purified EV-A71 virions (10 μg/ml) overnight and blocked the wells with 3% BSA in PBS. Then, spleen cells (5 × 10^5^) in RPMI-10% FBS were added and incubated overnight at 37°C. Following incubation, the plate was washed, and HRP-conjugated goat anti-mouse IgGs (1:000, Bethyl Laboratories, Inc.) or IgAs (1:000, Bethyl Laboratories, Inc.) was added. After incubating plates for 2 h, the HRP conjugate was removed and washed. Then, 100 μl of 3-amino-9-ethylcarbazole substrate (Sigma) was added and spots were allowed to develop in the dark at room temperature (RT) for 10 min. After ELISPOT analysis was completed, the plate was analyzed using an ImmunoSpot^®^ S6 UV Reader (Cellular Technology Limited, Cleveland, OH, United States). ImmunoSpot^®^ v.6.0 Software was used to automatically count the spots for each EV-A71 antigen stimulation condition and medium negative controls. Spots from a total of three wells with 1.5 × 10^6^ spleen cells were determined and considered as one set of data.

### T-Cell Response Analysis

Single cell suspensions from the spleens or Peyer’s patches of immunized mice were cultured in the presence or absence of 10 μg/ml of inactivated purified EV-A71 in RPMI-1640/10% FBS (35). For cytokine detection, cells were cultured at 37°C for 72 h, and supernatants were collected for IFN-γ, IL-17, and IL-4 assay (R&D Systems). To perform the proliferation test, splenocytes were cultured for 5 days and 1 μCi of [^3^H]-thymidine was added afterward for 18 h at 37°C. Then, cells were collected, and thymidine incorporation was detected using a scintillation counter (TopCount NXT Scintillation and Luminescence Counter; PerkinElmer).

### Flow Cytometry Analysis

Splenocytes were treated with EV-A71 for 5 days. After incubation, splenocytes were treated with phorbol 12-myristate 13-acetate (PMA), ionomycine, and monensin for 4 h. Then, cells were harvested and washed using the fluorescence activated cell sorting (FACS) buffer. After centrifugation, the Fc blocker were added to cells for 15 min at 4°C and cell surface antigens (CD4 and CD8) were stained for 30 min at 4°C. After washing, cells were fixed for 20 min at RT. Then, cells were washed and suspended in FACS buffer overnight at 4°C. To allow permeabilization, cells were washed twice with permeabilization buffer. Cells were blocked with Fc blocker for 15 min at 4°C and further stained with IFN-γ and IL-17 antibody for 60 min at RT. Finally, samples were washed and analyzed via flow cytometry (BD FACS Calibur).

### EV-A71 Neutralization Tests in Hybrid (hSCARB2^+/+^/Stat-1^–/–^) Mice

Serum samples obtained from immunized B6 mice 2 weeks after administering the third vaccination were co-incubated with 1 × 10^7^ pfu of live EV-A71 (genotype C2) for 2 h at 37°C (5 μl serum + 45 μl PBS + 50 μl EV-A71). After incubation, the mixture containing the serum and virus was intraperitoneally (i.p.) injected into 13-day-old hSCARB2^+/+^/*stat-1*^–/–^ mice (36). Survival rates were monitored daily post-injection for 20 days.

### RNA Extraction and Real-Time PCR

Hybrid hSCARB2^+/+^/stat-1^–/–^ mouse organs were homogenized in PBS buffer using protease inhibitors. The entire ribonucleic acid (RNA) content was extracted using TRIzol (Life technologies, United States) and chloroform (Merck Millipore). RNA precipitates were obtained using isopropanol (Merck Millipore) and 75% ethanol. For reverse transcription, complementary deoxyribonucleic acid (cDNA) was generated with the high-capacity cDNA RT kit (Life technologies; Biometra T 3000 Thermocycler). For amplifying DNA signals, the SYBR green supermix (Bio-Rad) was used to perform real-time polymerase chain reactions (Bio-Rad CFX connect). The forward and reverse cDNA sequences of primer pairs of GAPDH were as follows: forward: GTTCCTACCCCCAATGTG and reverse: CAACCTGGTCCTCAGTGTAG, and those for EV-A71 VP1 were as follows: forward: CTGGTAAAGGTCCAGCACTC and reverse: GGGAGGTCTATCTCTCCAAC. VP1 gene expression levels were normalized to those of GAPDH.

### Protective Immunity Against Live EV-A71 Challenge in hSCARB2-Tg Mice

The hSCARB2-Tg mouse was provided generously by Professor Satoshi Koike (16). This Tg mouse model is susceptible to EV-A71 infection. The hSCARB2-Tg mice were intranasally immunized with PBS (*n* = 10), 1 μg of EV-A71 alone (*n* = 9), or EV-A71 (1 μg) + PS-G (10 μg; *n* = 9) on day 7 and 14 of birth, and then intragastrically (i.g.) challenged with 1 × 10^7^ pfu of a mouse-adapted EV-A71/MP4 strain derived from the parental strain EV-A71/Tainan/4643/98 on day 21 (30). The clinical scores and survival rates of mice were observed daily for 2 weeks.

### Statistical Analysis

All data were expressed as mean ± SEM values, and analyzed and plotted using GraphPad Prism 7 Software. One-way ANOVA analysis and student’s *t*-test were used to compare results between different groups, and the statistically significance was defined as *P* < 0.05.

## Results

### EV-A71-Specific Antibody Responses to Intranasal EV-A71 Immunization Using PS-G as an Adjuvant

**FIGURE 1:**
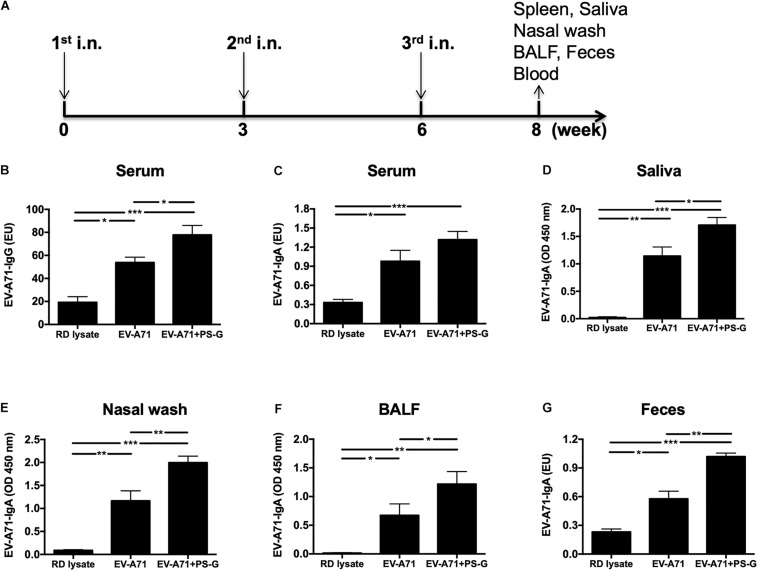
The effect of PS-G as an adjuvant in EV-A71-specific antibody response generation in immunized mice. Mice were intranasally immunized thrice with RD lysate, formalin-inactivated EV-A71 (2.5 μg/mouse), and formalin-inactivated EV-A71 (2.5 μg/mouse) plus PS-G (20 μg/mouse) at 3-week intervals. **(A)** Three-dose immunization schedules. Levels of EV-A71-specific IgG in the serum **(B)** and EV-A71-specific IgA in the serum **(C)**, saliva **(D)**, nasal wash **(E)**, BALF **(F)**, and feces **(G)** of mice were measured via ELISA after the third immunization. Similar results were obtained from three independent studies. *p < 0.05, **p < 0.01, and ***p < 0.001.

Mice were intranasally vaccinated thrice at 3-week intervals (Figure 1A) with RD lysate, 2.5 μg of formalin-inactivated EV-A71, and 2.5 μg of formalin-inactivated EV-A71 plus 20 μg of PS-G. In comparison to the RD lysate group, the use of EV-A71 alone, and EV-A71 combined with PS-G as an adjuvant generated a significant amount of EV-A71-IgG in the serum (Figure 1B), along with EV-A71-IgA in the serum, saliva, nasal wash, BALF, and feces, after the third immunization was administered (Figures 1C–G). When compared to EV-A71 group, the combination of EV-A71 with PS-G, used as an adjuvant, generated a significant amount of EV-A71-specific IgAs in the saliva (*p* < 0.05), nasal wash (*p* < 0.01), BALF (*p* < 0.05), and feces (*p* < 0.01) after the third vaccination (Figures 1D–G).

### Neutralization Titers in Serum and Saliva Following Intranasal EV-A71 Immunization Using PS-G as an Adjuvant

**FIGURE 2:**
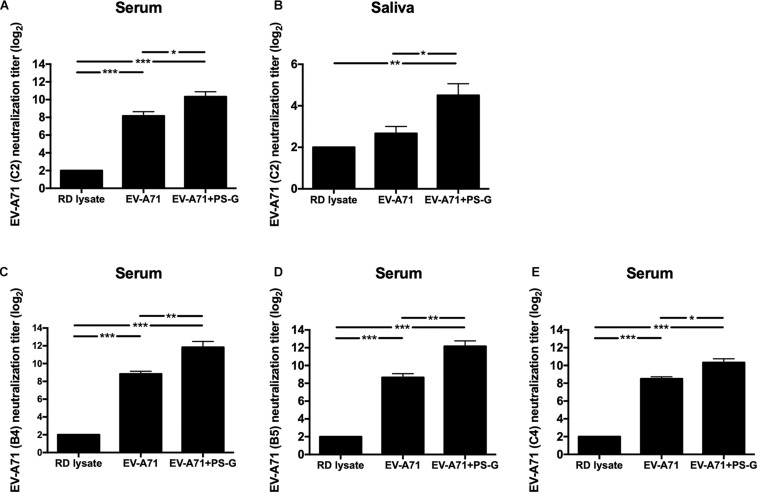
The effect of PS-G as an adjuvant on the EV-A71-specific neutralization titer against different EV-A71, **(A,B)** TW2272/98 (C2 genogroup), **(C)** 200307025 (B4 genogroup), **(D)** 20080738 (B5 genogroup), and **(E)** 200802571 (C4 genogroup). Sera and saliva were collected from mice, 2 weeks after the third immunization, and serially diluted (2^3–^2^12^), mixed with the different strains of EV-A71, and then used to infect RD cells. After incubation was performed for 4 days, the neutralization titer was decided as the highest dilution that generated no CPE in the virus. These results were based on three independent experiments. *p < 0.05, **p < 0.01, and ***p < 0.001.

Here, the EV-A71 vaccine was obtained from the TW2272/98 strain (C2 genogroup) circulating in 1998 (37); the neutralizing titer was detected by identifying the highest dilution that neutralized the TCID_50_ of TW2272/98 virus 100-fold, resulting in no cytopathic effects in EV-A71-sensitive RD cells. Mice vaccinated with RD lysate, EV-A71 alone, and EV-A71 + PS-G could generate antibodies in the serum, and were capable of neutralizing EV-A71 of the C2 genotype (Figure 2A). However, the neutralizing-antibody titer of mice vaccinated with the PS-G adjuvant EV-A71 was significantly higher than those of mice immunized with EV-A71 (*p* < 0.05). We also found that the neutralizing-antibody titer in saliva of mice immunized with PS-G adjuvant EV-A71 (Figure 2B) was significantly higher than that of mice immunized with RD lysate (*p* < 0.01) and EV-A71 (*p* < 0.05). To study the cross-reactivity of induced antibodies in the serum, we performed neutralization assays using three other strains of EV-A71: 200307025, the B4 genogroup, circulating in 2003 (Figure 2C); 20080738, the B5 genogroup, isolated in 2008 (Figure 2D); and 200802571, the C4 genogroup, isolated in 2008 (Figure 2E). The neutralization titers detected against the B4 genogroup were similar to those against the B5 and C4 genogroups. The EV-A71 + PS-G group resulted in a significantly higher cross neutralization titer than EV-A71 alone (*p* < 0.01 in B4 and B5 genogroup; *p* < 0.05 in C4 genogroup).

### Antibody-Secreting B Cells in the Spleen Following Intranasal EV-A71 Immunization Using PS-G as an Adjuvant

**FIGURE 3:**
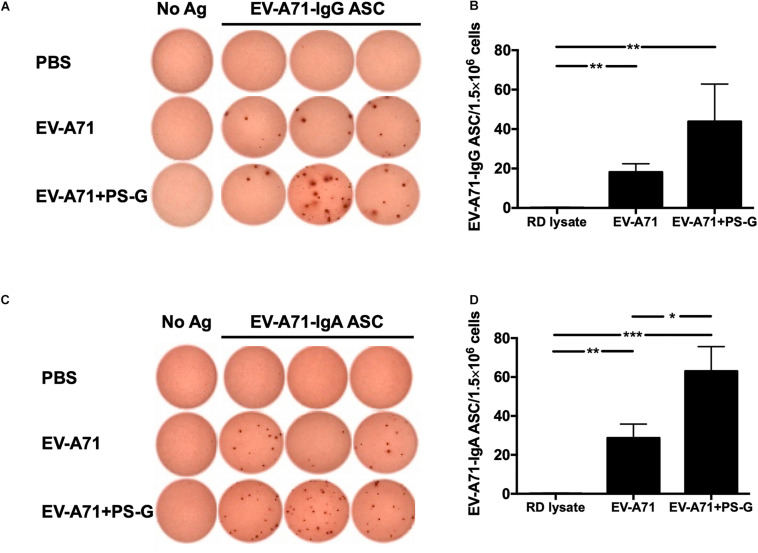
The effect of using PS-G as an adjuvant on the number of EV-A71-specific IgG antibody-secreting cells (ASCs) and IgA ASCs in splenocytes. Mice were intranasally immunized with RD lysate or formalin-inactivated EV-A71 with or without PS-G as an adjuvant for three times, at intervals of 3 weeks. The spleen was isolated 2 weeks after the third immunization was performed, and EV-A71-specific IgG ASCs **(A,B)** and IgA ASCs **(C,D)** were measured via ELISPOT. Similar results were obtained from three independent studies. *p < 0.05, **p < 0.01, and ***p < 0.001.

In order to evaluate the quality of memory responses caused by EV-A71 vaccine using PS-G as an adjuvant, we detected the level of antibody-secreting B cells (ASCs) in the splenocyte, 2 weeks after the third immunization. The extent of occurrence of EV-A71-specific IgG ASCs in the splenocytes was measured using ELISPOT. In comparison to the RD lysate group, the number of EV-A71 IgG ASCs were remarkably enhanced in groups of mice immunized using EV-A71 alone (*p* < 0.01) and EV-A71 plus PS-G (*p* < 0.01; Figures 3A,B); the number of EV-A71-specific IgA ASCs was significantly increased in mice groups vaccinated with EV-A71 alone (*p* < 0.01) and EV-A71 plus PS-G (*p* < 0.001; Figures 3C,D). The number of EV-A71-specific IgA ASCs was significantly increased in vaccinated mice belonging to the EV-A71 plus PS-G group, as compared to those from the EV-A71 immunized (*p* < 0.05; Figures 3C,D). The results suggested that as an adjuvant, PS-G induced EV-A71-specific IgG and IgA ASCs in the spleen more effectively.

### Effects of the PS-G Adjuvant on the Cellular Immune Responses in EV-A71-Vaccinated Mice

**FIGURE 4:**
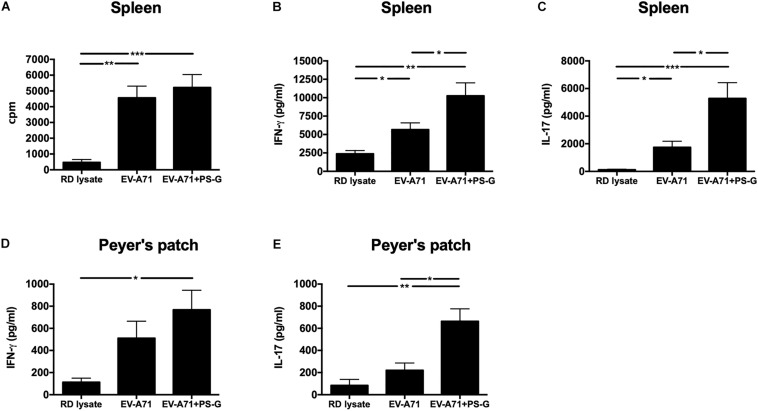
Proliferation and cytokine levels of splenocytes and Peyer’s patch (PP) cells derived from immunized mice. Splenocytes and Peyer’s patch cells from mice immunized with RD lysate, formalin-inactivated EV-A71, or adjuvanted with PS-G were collected and cultured in a RPMI medium containing 10 μg/ml of heat-inactivated EV-A71. **(A)** Cells were cultured for 5 days, after which proliferation was measured as the [^3^H]-thymidine level incorporated in splenocytes. Culture supernatants of splenocytes and Peyer’s patch cells were detected for IFN-γ **(B,D)** and IL-17 production **(C,E)** after culturing for 3 days with heat-inactivated EV-A71. Thymidine uptake and incorporation were analyzed by harvesting cells using a scintillation counter to detect incorporation levels (counts per minute; cpm). Similar results were obtained from three separate experiments. *p < 0.05, **p < 0.01, and ***p < 0.001.

**FIGURE 5:**
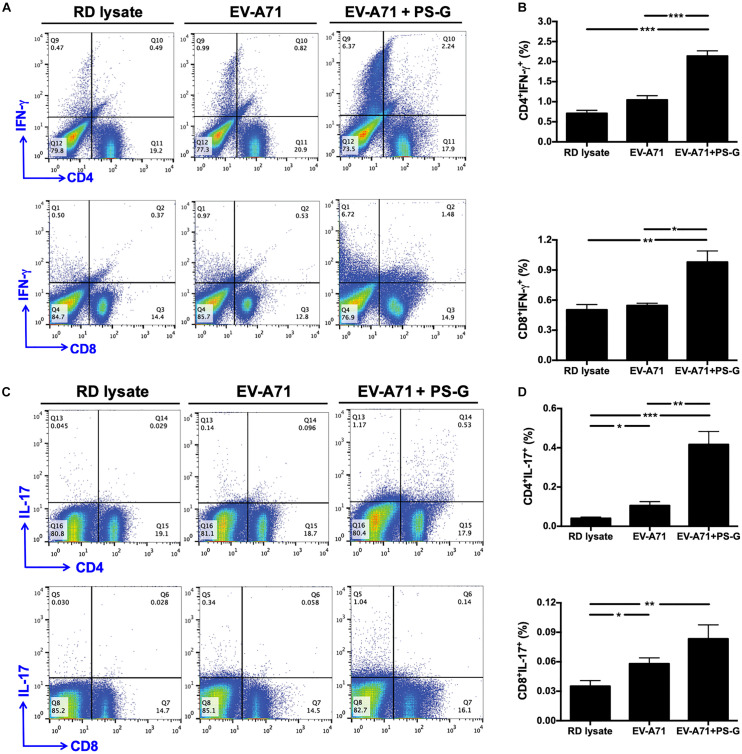
IFN-γ and IL-17 were produced by CD4^+^ and CD8^+^ T cells in the spleen and subsequently obtained from immunized mice. The splenocytes from immunized mice with RD lysate, formalin-inactivated EV-A71, or adjuvanted with PS-G were collected and cultured in a RPMI medium containing 10 μg/ml of heat-inactivated EV-A71. **(A–D)** Cells were cultured for 5 days; levels of IFN-γ and IL-17 produced by CD4^+^ and CD8^+^ T cells were measured using flow cytometry. Results from one out of three representative experiments are shown. *p < 0.05, **p < 0.01, and ***p < 0.001.

The intensity of the helper T cell responses plays a major role in generating both cellular and humoral immune responses. The result indicated that T cell proliferation and activation was induced in response to EV-A71 antigen in groups of mice immunized with EV-A71 and EV-A71 + PS-G (Figure 4A). The levels of cytokines produced by splenocytes were detected as an indicator of the memory T cells response. The levels of IFN-γ, IL-17, and IL-4 produced by splenocytes stimulated with the EV-A71 antigen were measured via ELISA. When compared to the RD lysate group, splenocytes from the mouse group vaccinated with EV-A71 + PS-G produced remarkably higher levels of IFN-γ (*p* < 0.01; Figure 4B) and IL-17 (*p* < 0.001; Figure 4C). When compared to the EV-A71 group, the mice group vaccinated with EV71 + PS-G induced remarkably higher levels of IFN-γ and IL-17 (*p* < 0.05; Figures 4B,C) in splenocytes after EV-A71 antigen stimulation. We also analyzed cytokine levels produced when Peyer’s patch cells were stimulated with the EV-A71 antigen. When compared to the RD lysate group, the formulation containing EV-A71 and PS-G caused a significant amount of IFN-γ (*p* < 0.05) to be secreted in Peyer’s patch cells after the third vaccination was administered (Figure 4D). In Figure 5E, we found that the EV-A71 + PS-G group released a significant amount of IL-17 from Peyer’s patch cells after the third vaccination was administered, as compared to that released by other groups. However, the IL-4 level was undetectable in the splenocytes and Peyer’s patch cells of all mice (data not shown). In this study, we found that PS-G could enhance adaptive Th1 and Th17 responses, but not the Th2 response.

### Induction of IFN-γ and IL-17 by CD4^+^ and CD8^+^ T Cells in the Spleen Following Intranasal EV-A71 Immunization Using PS-G as an Adjuvant

In order to understand the immune activation mechanism via the EV-A71 mucosal vaccine more effectively, the systemic response of CD4^+^ and CD8^+^ T cells in vaccinated mice was investigated. Splenocytes from mice immunized with RD lysate, formalin-inactivated EV-A71, or adjuvanted with PS-G were collected and treated with 10 μg/ml of heat-inactivated EV-A71 in 10% FBS-RPMI medium. Cells were cultured for 3 days, and IFN-γ and IL-17 levels produced by CD4^+^ and CD8^+^ T cells were measured using flow cytometry (Figures 5A–D). We confirmed that significant levels of IFN-γ were released by CD4^+^ and CD8^+^ T cells in spleen of EV-A71 + PS-G-vaccinated mice (Figures 5A,B). We further attempted to identify the cells that could secrete IL-17. We found that CD4^+^ and CD8^+^ T cells could secret IL-17; CD4^+^ T cells produced a higher level of IL-17 than CD8^+^ T cells in EV-A71 + PS-G-vaccinated mice (Figures 5C,D). These data demonstrated that both CD4^+^ and CD8^+^ T cells could be involve in EV-A71 vaccine-mediated antiviral protection.

### EV-A71 Neutralization Test in Hybrid (hSCARB2^+/+^/Stat-1^–/–^) Mice

**FIGURE 6:**
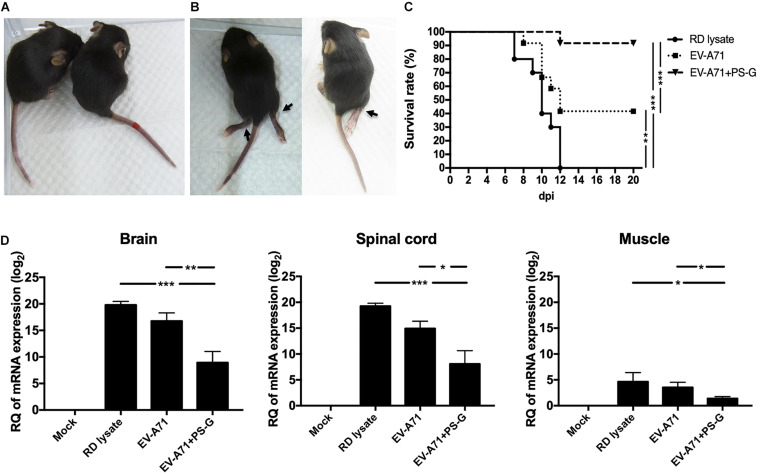
Results of the EV-A71 neutralization test in hybrid (hSCARB2^+/+^/stat-1^–/–^) mice. Serum samples from immunized B6 mice (including RD lysate, EV-A71, and EV-A71 + PS-G-immunized group) were co-incubated with 1 × 10^7^ pfu EV-A71 for 2 h at 37°C. After incubation, 13-day-old newborns of hybrid mice (hSCARB2^+/+^/stat-1^–/–^) were i.p. infected with EV-A71 and the serum mixture. **(A)** Representative images showing protected mice in the EV-A71 or EV-A71 + PS-G immunized group of mice with healthy limbs and **(B)** the RD lysate-immunized group of mice exhibiting limb paralysis at day 7 post-infection. **(C)** Survival rates were monitored daily post-injection for 20 days. **(D)** The brain, spinal cord, and muscles were collected after infection and viral loads within tissues were detected using real-time quantitative RT-PCR. Data were normalized with those for GAPDH. *p < 0.05, **p < 0.01, and ***p < 0.001.

The hybrid hSCARB2^+/+^/stat-1^–/–^ mouse strain was most susceptible to EV-A71 infections and illness (36). Serum samples from RD lysate, EV-A71, or EV-A71 + PS-G-immunized B6 mice were co-incubated with 1 × 10^7^ pfu EV-A71 (genotype C2) for 2 h at 37°C. After incubation, 13-day-old newborns of hybrid (hSCARB2^+/+^/stat-1^–/–^) mice were injected intraperitoneally with a mixture of EV-A71 and serum. Representative images show protected mice in the EV-A71 or EV-A71 + PS-G immunized groups with healthy limbs (Figure 6A) and the RD lysate-immunized group with limb paralysis (Figure 6B), 7 days post-infection. Survival rates were monitored daily post-injection for 20 days. On day 20 after the injections was administered, survival rates in the EV-A71 and EV-A71 + PS-G groups were 42% (*p* < 0.01) and 92% (*p* < 0.001), respectively, while none of the mice in the RD lysate group survived (Figure 6C). To verify the replication and distribution of EV-A71 in infected mice, the viral loads of EV-A71-infected mice in various tissues (brain, spinal cord, and muscle) were measured via real-time PCR (Figure 6D). Viral loads were significantly higher in the brain and spinal cord than in the muscle. EV-A71 VP1-specific RNA in the brain, spinal cord, and muscle of mice in the EV-A71 + PS-G group were significantly decreased. These findings indicated that the EV-A71 + PS-G vaccine has considerable protective effects against an EV-A71 infection.

### Protective Immunity Against Live EV-A71 Challenge in hSCARB2-Tg Mice

**FIGURE 7:**
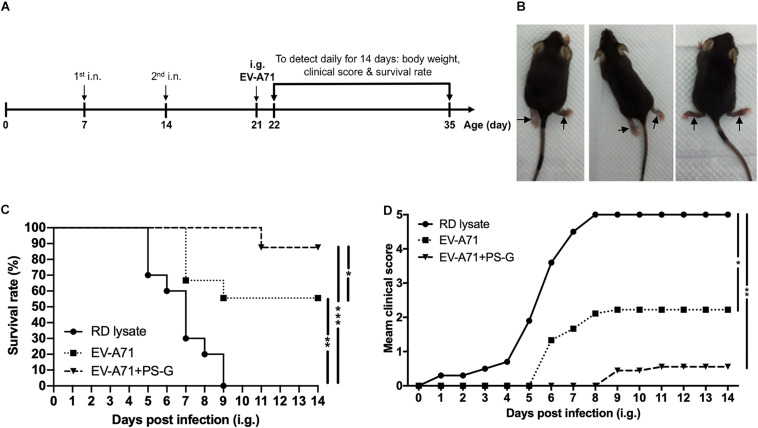
Through immunization with the EV-A71 mucosal vaccine, hSCARB2-Tg mice were protected from live EV-A71 challenges. The hSCARB2-Tg mice were intranasally immunized twice with RD lysate, EV-A71 (1 μg), or EV-A71 (1 μg) with PS-G (10 μg) on days 7 and 14 before they were i.g. challenged with 1 × 10^7^ pfu of live EV-A71. **(A)** Two-dose immunization and challenge schedule. **(B)** Representative mice with limb paralysis on day 8 after the administration of MP4. After 21-day-old hSCARB2-Tg mice were i.g. infected with EV-A71, **(C)** the daily survival rates and **(D)** clinical scores were monitored. The clinical score was defined as: 0, healthy; 1, wasting; 2, weak limbs; 3, paralysis in only one limb; 4, paralysis in two to four limbs; and 5, death. *p < 0.05, **p < 0.01, and ***p < 0.001.

The hSCARB2-Tg mouse is a helpful model for evaluating anti-EV-A71 drugs and studying EV-A71 induced pathogenesis (16). hSCARB2-Tg mice were intranasally immunized twice with RD lysate alone, EV-A71, and EV-A71 + PS-G on day 7 and 14 after birth, and were then intragastrically challenged with 1 × 10^7^ pfu of a mouse-adapted EV-A71/MP4 strain on day 21 after birth (Figure 7A), and subsequently monitored on a daily basis to identify its clinical manifestation and survival rate. Figure 7B shows representative mice with limb paralysis. The result demonstrated that in the EV-A71 and EV-A71 + PS-G immunized groups, the survival rates of mice were 56% (*p* < 0.01) and 89% (*p* < 0.001), respectively (Figure 7C). The clinical scores of the EV-A71 + PS-G group were significantly lower than those of the EV-A71 (*p* < 0.05) and RD lysate (*p* < 0.01) groups (Figure 7D).

## Discussion

The mucosal surface is the major site of entry of most pathogens, and the effective induction of mucosal immunity is the first line of defense of the host against invading pathogens (38). Here, we found that intranasal administration of formalin-inactivated EV-A71, using PS-G as an adjuvant generated high titers of anti-EV-A71 IgA in the saliva, nasal wash, BALF, and feces, and induced anti-EV-A71 IgG and IgA production in the serum. Importantly, these antibodies could neutralize the different EV-A71 genotypes infections. Vaccination via the nasal mucosal has been widely studied (39), because the nasal route is easily accessible, simpler, cheaper, and does not require needles. The nasal cavity contains a large number of DCs, B cells, and T cells, they are covered by an epithelial layer containing unique cells named microfold cells (40). Here, we found that intranasal immunization induced EV-A71-specific IgAs not only in the respiratory tract, but also in other mucosal sites (for example the intestine). The mucosal surface is largely distributed in the human body, and different parts of the mucosal surface can be connected to each other via lymphocyte homing, which causes immunization to occur at a distant mucosal effector site. The combination of formalin-inactivated EV-A71 and PS-G is considered to be an excellent vaccine, as it induces high titers of neutralizing antibodies that resist lethal viral infections. It could also enhance the secretory-IgA antibody production, which proves that immunization through the mucosal pathway could produce both mucosal and systemic immune responses.

In recent years, East and Southeast Asian countries have witnessed several large-scale outbreaks of HFMD, owing to different subgenotypes of EV-A71 (1, 41). Thus, for EV-A71 mucosal vaccine development, cross-protection against other EV-A71 genotypes is crucial. The sequence of VP1, a key antigen, varies greatly among enteroviruses. EV-A71 can be divided into several genogroups based on their VP1 gene sequences. Here, the EV-A71 C2 genotype-based formalin-inactivated mucosal vaccine could enhance the production of several antibodies that not only resist the EV-A71 C2 genogroup, but also resist the B4, B5, and C4 genogroups, demonstrating that the vaccine exhibited broad levels of cross-protection against EV-A71 strains with different genotypes.

During immunogenicity assessments, antibody-secreting cells could be used as a key indicator of immune memory on the mucosal surface. We found that there were more EV-A71-IgG and -IgA antibody-secreting cells in splenocytes of the group immunized with PS-G-adjuvanted EV-A71. These results corelated with EV-A71-specific IgG and IgA antibody titers in the serum and mucosal tissue, suggesting that the vaccine containing EV-A71 and PS-G could stimulate B lymphocyte proliferation and induce humoral immunity. In addition, with regard to T lymphocyte immunity, T-cell proliferation, IFN-γ and IL-17 production were significantly enhanced in splenocytes when EV-A71 was formulated with PS-G as an adjuvant. We also found that the levels of IFN-γ and IL-17 released in Peyer’s patch cells were significantly increased when EV-A71 was formulated with PS-G, used as an adjuvant. The results suggested that EV-A71 mucosal immunization greatly stimulated Th1- and Th17-type cytokine production. We further demonstrated that CD4^+^ and CD8^+^ T cells could produce IFN-γ and IL-17 in splenocytes with flow cytometry analysis. These results demonstrated that the number of CD4^+^ and CD8^+^ T cells were improved in the EV-A71 + PS-G group. The activation of EV-A71 antigen-specific CD4^+^ and CD8^+^ T cells is a major indicator of the cellular immune response. Both CD4^+^ and CD8^+^ T cells reportedly protected mice from EV-A71 infections, by reducing viral loads in tissues (42). The CD8^+^ T cells have antigen-specific cytotoxic activity; they can alter self-cells by mounting a cytotoxic reaction that lyses virus-infected cells. In our lethal infection study, SCARB2-Tg mice immunized with two doses of EV-A71 formulated with PS-G as an adjuvant could protect against a lethal EV-A71 intragastrical challenge. Our results indicated that EV-A71 mucosal immunization could induce an effective T cells immune responses, thereby helping to clear the EV-A71 virus, and protecting mice from EV-A71 infection. Taken together, these data suggested that the use of PS-G as an adjuvant in the EV-A71 nasal vaccine could enhance potential immune cell responses.

In this study, we used two animal models, which included a hybrid mouse model (STAT-1 KO and hSCARB2-Tg) and a hSCARB2-Tg mouse model, to confirm whether the PS-G formulated vaccine conferred *in vivo* protection against EV-A71 challenge. The rationale for selecting the hybrid mouse model is that it is highly susceptible to inoculation at a lower viral dose (36). Furthermore, this hybrid mouse model is an easy-accessed and can be infected even at approximately 2 weeks after birth. For the i.p. infection of the hybrid mice model, clinical isolates (with no adaptation through serial passages) were used. For the i.g. infection model with hSCARB2-Tg mice (16) used in this study, we relied on the use of a mouse-adapted MP4 strain of EV-A71, and the model could be infected at 3 weeks after birth. Therefore, these two models are complementary to each other.

Emerging vaccination mechanisms propose that DCs play a critical role in determining the magnitude of the immune reactions. The use of antigens alone could usually not induce a strong response, and the addition of an appropriate adjuvant is a widely used strategy for enhancing the immunogenicity of vaccine antigens. Reishi has reportedly shown effective results in immune function modulation. The antitumor activity of polysaccharides isolated from *G. lucidum* was reportedly attributed to a structure with a branched glucan core comprising (1→3)-β-, (1→4)-β-, and (1→6)-β-linkages (24). Biological studies on *G. lucidum* have shown that PS-G performs multiple biological activities, including enhancement of cytotoxic activity of natural killer cells, enhancement of TNF-α and IFN-γ release from macrophages and lymphocytes, respectively, (26) and promotion of neutrophil lifespan; these actions depend on the activation of Akt-regulated signaling pathways (43). It also induces the migration and phagocytic activity of human primary neutrophils, and further provide evidence to strengthen the beneficial effects of PS-G in humans to enhance the defense system (44). In previous studies, we have demonstrated that PS-G could induce the activation and maturation of human monocyte-derived DCs through NF-kB and p38 mitogen-activated protein kinase pathways (28), and that PS-G could stimulate DCs through TLR4 to induce IL-12 secretion, which led to the significant stimulation of T lymphocyte growth and differentiation. In immune responses, IL-12 plays a central role as a link between the innate and adaptive immune systems (45, 46). In addition, IL-12 polarizes the immune system toward a primary Th1 response. Additionally, we found that PS-G could induce the production of Th1-related cytokines and chemokines in DCs, and also acted as an adjuvant-active molecule that could stimulate the Th1 immune response and antibody production, as observed in an *in vivo* study (29). The induction of DC maturation is critical for the induction of antigen-specific T lymphocyte responses, and may be essential for the development of human vaccines that rely on T cell immunity. Hsu et al. also has demonstrated that an extract of *G. lucidum* polysaccharides could induce IL-1 expression via TLR4-modulated protein kinase signaling pathways in macrophages (47). Zhang et al. also has reported that the *G. lucidum* polysaccharide acts as an adjuvant for chicken Newcastle disease vaccine (48). Here, we found that PS-G could effectively induce EV-A71-specific immune responses via the intranasal route. PS-G could function as an adjuvant based on their effects on antigen-presenting cells and the induction of immunomodulatory cytokines.

The main purpose of intranasal vaccine development is to design an adjuvant that could ensure valid mucosal immune activity, and is stable and safe for clinical application in humans. Cholera and heat-labile *E. coli* toxins have been used as adjuvants to induce the mucosal immune response (49). These toxins effectively provoke mucosal immune responses; however, in the following year, their correlation with facial nerve paralysis was reported (20). Poly (I:C) is also an effective mucosal adjuvant; however, it is associated with certain adverse events in humans. PS-G is derived from the fruiting bodies of *G. lucidum* and is extracted via boiling, which indicates that the active ingredients in PS-G are heat resistant, whereas poly (I:C) loses its adjuvant activity when boiled for 5 min at 95°C (50). The PS-G sample alone and in combination with EV-A71 did not induce significant changes in biological activity during the experiment, and no change was observed in the physical state (such as no precipitation or discoloration) either. We are aware that the acidity in the stomach environment may generate partial acid hydrolysis products from polysaccharides. The aqueous solutions of PS-G and antigen were prepared by dissolving them in PBS at the physiological pH of 7.4. PS-G was mixed gently with the antigen and administered via the nasal route in this study. Therefore, we believe that PS-G alone and in combination with EV-A71 remain stable under physiological conditions. On the other hand, the toxicity test is important to make comprehensive evaluation on its safety. Here, we demonstrated that PS-G had no obvious cytotoxicity effect on DCs (Supplementary Figure 1), and did not show considerable local or peripheral immune toxicity in C57BL/6 (6-14-week-old) and hSCARB2-Tg (1-5-week old) mice. In the future, the rigor pharmacology study of PS-G will be applied to the mechanism of its immune-regulation activity, as well as the potential in vaccine development as adjuvants.

In conclusion, these results demonstrated that the use of PS-G as an adjuvant in the EV-A71 nasal vaccine generated a powerful mucosal immune response. Thus, the used of PS-G as a safe and effective adjuvant is recommended for intranasal EV-A71 mucosal vaccine development.

## Data Availability Statement

All datasets presented in this study are included in the article/Supplementary Material.

## Ethics Statement

The animal study was reviewed and approved by the Institutional Animal Care and Use Committee (Approval No: 20130272) at the College of Medicine, National Taiwan University (Taipei, Taiwan).

## Author Contributions

Y-LL and B-LC conceived this research, supervised the project, analyzed the data, and wrote the manuscript. Y-LL, CS, P-YC, C-LC, A-TL, and P-YL performed the experiments, collected data, analyzed the data, and performed statistical analysis. All authors have read and agreed to the submitted version of the manuscript.

## Conflict of Interest

P-YL was employed by Good Health Food Co., Ltd. The remaining authors declare that the research was conducted in the absence of any commercial or financial relationships that could be construed as a potential conflict of interest.

## Supplementary Material

Supplementary Figure 1 NMR spectrum for PS-G composition. The mannose naphthimidazole (Man-NAIM) derivative is indicated in **(A)**. The glucose naphthimidazole (Glc-NAIM) derivative is indicated in **(B)**. PS-G was hydrolyzed and labeled with naphthalene diamine, as indicated in **(C)**. PS-G was primarily composed of glucose (79%) and mannose (21%) from the integration in **(C)**.

Supplementary Figure 2 2D DOSY NMR spectrum for PS-G. The molecular weight of PS-G was about 15 kDa. Evaluation of cell viability by CCK-8 assay. The harvested DCs were plated at 1 × 10^5^ cells per well in a 96-well plate and treated with various concentrations of PS-G (0.2, 2, 20, and 200 μg/ml) for 24 h. Subsequently, 10% CCK-8 was added to each well and the cells were incubated for 2 h at 37°C in dark, and the optical density (OD) was measured at 450 nm was determined using a SpectraMax M5 Multi-Mode Microplate Reader (Molecular Devices). The average viability of control cells was set at 100%, and the resultant cell viabilities were expressed as a percentage of this value. Cytotoxicity of PS-G in DCs. Mouse bone marrow cells were differentiated into dendritic cells (DCs) by resuspension in complete medium RPMI-1640 supplemented with 10% fetal bovine serum, 10 ng/ml interleukin-4 (IL-4), and 10 ng/ml granulocyte-macrophage colony-stimulating factor (GM-CSF) for 6 days, following which the dendritic extensions were observed under an optical microscope. The viabilities of DCs that were treated with different concentrations of PS-G (0.2, 2, 20, and 200 μg/ml) for 24 h were estimated in the Cell Counting Kit-8 (CCK-8) assay. The result showed that PS-G did not exert any obvious cytotoxic effects on DCs at the concentrations mentioned above (Supplementary Figure 3).

Supplementary Figure 3 Analysis of PS-G cytotoxicity on DCs. The cytotoxicity effects of PS-G on DCs were evaluated by the CCK-8 assay. Cells were treated with different concentrations of PS-G, as indicated, for 24 h. The optical density was measured at 450 nm using a Microplate Reader. The average viability of control cells was considered as 100%, and the resultant viabilities were expressed as a percentage of this value. Data are expressed in terms of mean ± SEM from three independent experiments. Immunization of mice. SPF female C57BL/6 mice (6-week-old) were used to study the effects of different doses of PS-G as an adjuvant on the immune response to EV-A71. Six mice from each group were immunized intranasally with the vaccine, which included RD lysate, 2.5 μg of formalin-inactivated EV-A71, 2.5 μg of formalin-inactivated EV-A71 plus 2 μg of PS-G, and 2.5 μg of formalin-inactivated EV-A71 plus 20 μg of PS-G as an adjuvant. The mice were inoculated thrice on days 0, 21, and 42. Blood, saliva, and fecal specimens were collected at 2 weeks after the third immunization process and stored at −80°C until further use. EV-A71-specific antibody responses to intranasal EV-A71 immunization with different doses of PS-G as an adjuvant. The mice were vaccinated intranasally thrice at 3-week intervals with RD lysate, 2.5 μg of formalin-inactivated EV-A71, and 2.5 μg of formalin-inactivated EV-A71 plus 2 μg or 20 μg of PS-G. In comparison to the RD lysate group, the groups treated with EV-A71 alone, or with EV-A71 plus 2 μg or 20 μg of PS-G as an adjuvant showed the expression of EV-A71-IgG at significant levels in the serum (Supplementary Figure 4A), along with EV-A71-IgA expression in the saliva and feces (Supplementary Figures 4B,C), after the third immunization. Compared to EV-A71 group, the combination of EV-A71 with 20 μg of PS-G led to the production of EV-A71-specific IgG at significant levels in the serum (*p* < 0.01) and EV-A71-specific IgA in the saliva (*p* < 0.05) and feces (*p* < 0.01) compared to those in mice immunized with EV-A71 plus 2 μg of PS-G as an adjuvant after the third vaccination. Based on these results, we selected 20 μg of PS-G as the optimal adjuvant dose for intranasal immunization.

Supplementary Figure 4 The effect of different doses of PS-G as an adjuvant on EV-A71-specific antibody response generation in immunized mice. The mice were intranasally immunized thrice with RD lysate, formalin-inactivated EV-A71 (2.5 μg/mouse), and formalin-inactivated EV-A71 plus PS-G (2 μg or 20 μg/mouse) at 3-week intervals. The titer of EV-A71-specific IgG in the serum **(A)** and of EV-A71-specific IgA in the saliva **(B)**, and feces **(C)** of mice were measured via ELISA after the third immunization. **p* < 0.05, ***p* < 0.01, and ****p* < 0.001.
