# Supplementary material for: A Polysaccharide Purified From Ganoderma lucidum Acts as a Potent Mucosal Adjuvant That Promotes Protective Immunity Against the Lethal Challenge With Enterovirus A71
Source: Front Immunol. 2020 Sep 29;11:561758. doi: 10.3389/fimmu.2020.561758 (PMC7550786; doi:10.3389/fimmu.2020.561758)
Supplement: Supplementary Figure 1 — NMR spectrum for PS-G composition. The mannose naphthimidazole (Man-NAIM) derivative is indicated in (A). The glucose naphthimidazole (Glc-NAIM) derivative is indicated in (B). PS-G was hydrolyzed and labeled with naphthalene diamine, as indicated in (C). PS-G was primarily composed of glucose (79%) and mannose (21%) from the integration in (C). [file Data_Sheet_1.docx]

**Supplementary Materials:**


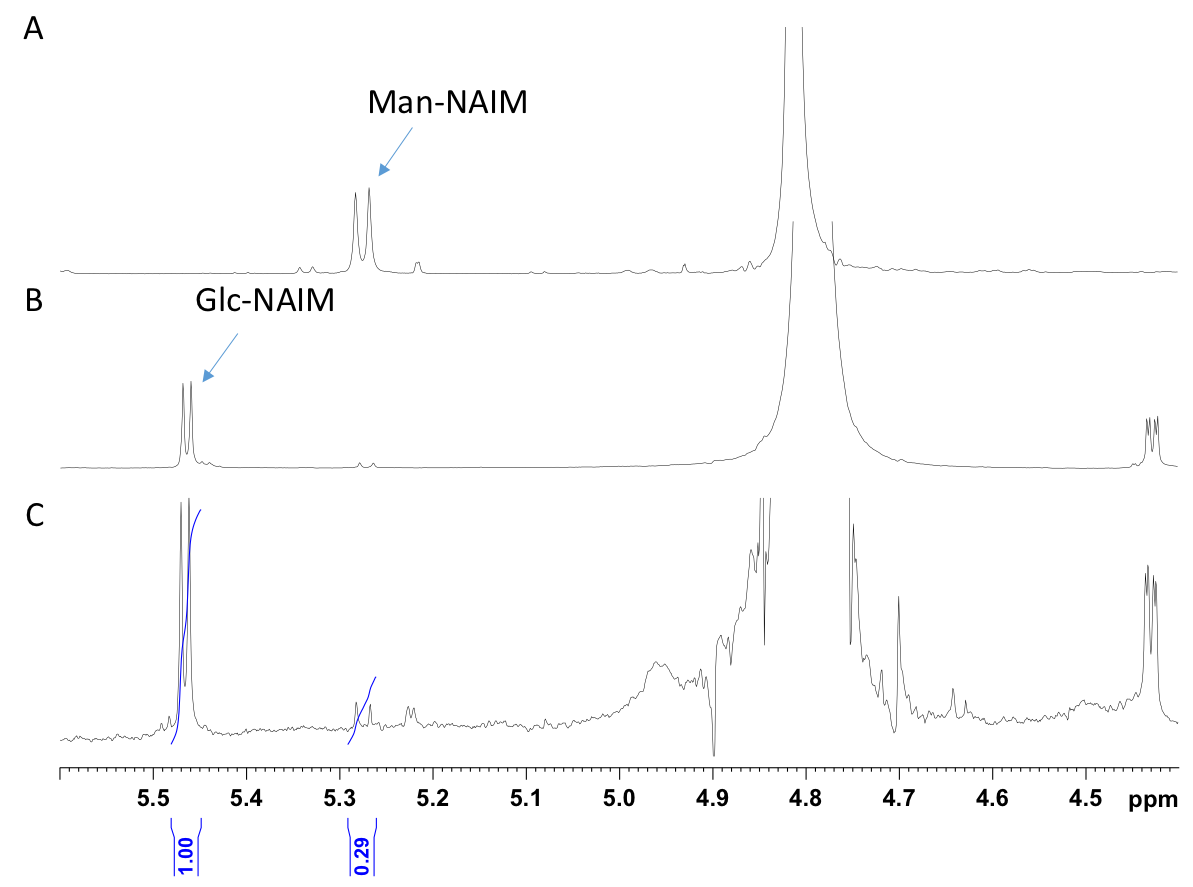


**Supplementary Figure 1. NMR spectrum for PS-G composition.** The mannose naphthimidazole (Man-NAIM) derivative is indicated in **(A)**. The glucose naphthimidzaole (Glc-NAIM) derivative is indicated in **(B)**. PS-G was hydrolyzed and labeled with naphthalene diamine, as indicated in **(C)**. PS-G was primarily composed of glucose (79 %) and mannose (21 %) from the integration in **(C)**.


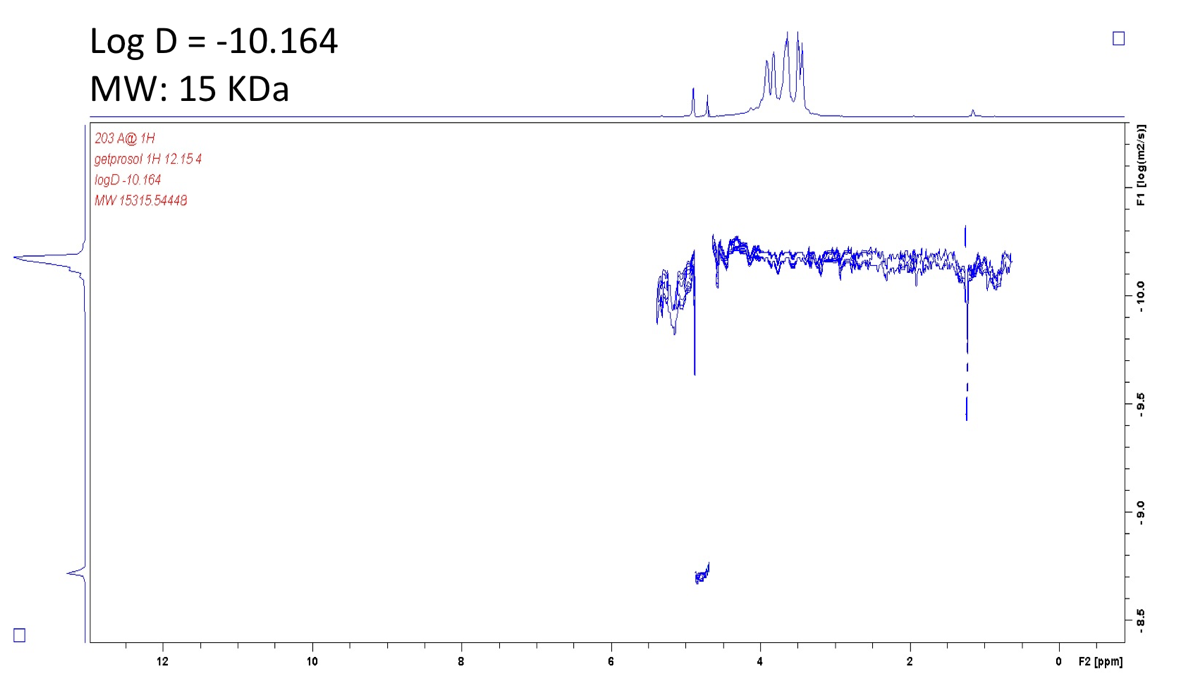


**Supplementary Figure 2. 2D DOSY NMR spectrum for PS-G.** The molecular weight of PS-G was about 15 kDa.

**Supplementary Figure 3. Analysis of PS-G cytotoxicity on DCs.** The cytotoxicity effects of PS-G on DCs were evaluated by the CCK-8 assay. Cells were treated with different concentrations of PS-G, as indicated, for 24 h. The optical density was measured at 450 nm using a Microplate Reader. The average viability of control cells was considered as 100 %, and the resultant viabilities were expressed as a percentage of this value. Data are expressed in terms of mean ± SEM from three independent experiments.

**Evaluation of cell viability by CCK-8 assay**

The harvested DCs were plated at 1×10^5^ cells per well in a 96-well plate and treated with various concentrations of PS-G (0.2, 2, 20, and 200 μg/ml) for 24 h. Subsequently, 10 % CCK-8 was added to each well and the cells were incubated for 2 h at 37 °C in dark, and the optical density (OD) was measured at 450 nm was determined using a SpectraMax M5 Multi-Mode Microplate Reader (Molecular Devices). The average viability of control cells was set at 100 %, and the resultant cell viabilities were expressed as a percentage of this value.

**Cytotoxicity of PS-G in DCs**

Mouse bone marrow cells were differentiated into dendritic cells (DCs) by resuspension in complete medium RPMI-1640 supplemented with 10 % fetal bovine serum, 10 ng/ml interleukin-4 (IL-4), and 10 ng/ml granulocyte-macrophage colony-stimulating factor (GM-CSF) for 6 days, following which the dendritic extensions were observed under an optical microscope. The viabilities of DCs that were treated with different concentrations of PS-G (0.2, 2, 20, and 200 μg/ml) for 24 h were estimated in the Cell Counting Kit-8 (CCK-8) assay. The result showed that PS-G did not exert any obvious cytotoxic effects on DCs at the concentrations mentioned above (**Supplementary Figure 3**).

**Supplementary Figure 4. The effect of different doses of PS-G as an adjuvant on EV-A71-specific antibody response generation in immunized mice.** The mice were intranasally immunized thrice with RD lysate, formalin-inactivated EV-A71 (2.5 μg/mouse), and formalin-inactivated EV-A71 plus PS-G (2 μg or 20 μg/mouse) at 3-week intervals. The titer of EV-A71-specific IgG in the serum **(A)** and of EV-A71-specific IgA in the saliva **(B)**, and feces **(C)** of mice were measured via ELISA after the third immunization. **p* < 0.05, ***p* < 0.01, and ****p* < 0.001.

**Immunization of mice**

SPF female C57BL/6 mice (6-week-old) were used to study the effects of different doses of PS-G as an adjuvant on the immune response to EV-A71. Six mice from each group were immunized intranasally with the vaccine, which included RD lysate, 2.5 μg of formalin-inactivated EV-A71, 2.5 μg of formalin-inactivated EV-A71 plus 2 μg of PS-G, and 2.5 μg of formalin-inactivated EV-A71 plus 20 μg of PS-G as an adjuvant. The mice were inoculated thrice on days 0, 21, and 42. Blood, saliva, and fecal specimens were collected at two weeks after the third immunization process and stored at -80 °C until further use.

**EV-A71-specific antibody responses to intranasal EV-A71 immunization with different doses of PS-G as an adjuvant**

The mice were vaccinated intranasally thrice at three-week intervals with RD lysate, 2.5 μg of formalin-inactivated EV-A71, and 2.5 μg of formalin-inactivated EV-A71 plus 2 μg or 20 μg of PS-G. In comparison to the RD lysate group, the groups treated with EV-A71 alone, or with EV-A71 plus 2 μg or 20 μg of PS-G as an adjuvant showed the expression of EV-A71-IgG at significant levels in the serum (**Supplementary Figure 4A**), along with EV-A71-IgA expression in the saliva and feces (**Supplementary Figure 4B, C**), after the third immunization. Compared to EV-A71 group, the combination of EV-A71 with 20 μg of PS-G led to the production of EV-A71-specific IgG at significant levels in the serum (p < 0.01) and EV-A71-specific IgA in the saliva (p < 0.05) and feces (p < 0.01) compared to those in mice immunized with EV-A71 plus 2 μg of PS-G as an adjuvant after the third vaccination. Based on these results, we selected 20 μg of PS-G as the optimal adjuvant dose for intranasal immunization.
